# Potency of Vaborbactam Is Less Affected than That of Avibactam in Strains Producing KPC-2 Mutations That Confer Resistance to Ceftazidime-Avibactam

**DOI:** 10.1128/AAC.01936-19

**Published:** 2020-03-24

**Authors:** Ruslan Tsivkovski, Olga Lomovskaya

**Affiliations:** a Qpex BioPharma, Inc., San Diego, California, USA

**Keywords:** vaborbactam, beta-lactamase inhibitors, ceftazidime-avibactam, KPC-2, mutations

## Abstract

Resistance to ceftazidime-avibactam due to mutations in KPC genes has been reported both *in vitro* and in clinical settings. The most frequently reported mutation leads to the amino acid substitution D179Y in the Ω loop of the enzyme. Bacterial cells that carry mutant KPC acquire a higher level of ceftazidime resistance, become more sensitive to other cephalosporins, and almost completely lose resistance to carbapenems. In this study, we demonstrated that two substitutions in KPC-2, D179Y and L169P, reduce the ability of avibactam to enhance the activity of ceftazidime, cefepime, or piperacillin against isogenic efflux-deficient strains of Pseudomonas aeruginosa, 8- to 32-fold and 4- to 16-fold for the D179Y and L169P variants, respectively, depending on the antibiotic. In contrast, the potency of vaborbactam, the structurally unrelated β-lactamase inhibitor that was recently approved by the FDA in combination with meropenem, is reduced no more than 2-fold. Experiments with purified enzymes demonstrate that the D179Y substitution causes an ∼20-fold increase in the 50% inhibitory concentration (IC_50_) for inhibition of ceftazidime hydrolysis by avibactam, versus 2-fold for vaborbactam, and that the L169P substitution has an ∼4.5-fold-stronger effect on the affinity for avibactam than for vaborbactam. In addition, the D179Y and L169P variants hydrolyze ceftazidime with 10-fold and 4-fold-higher efficiencies, respectively, than that of wild-type KPC-2. Thus, microbiological and biochemical experiments implicate both decreased ability of avibactam to interact with KPC-2 variants and an increase in the efficiency of ceftazidime hydrolysis in resistance to ceftazidime-avibactam. These substitutions have a considerably lesser effect on interactions with vaborbactam, making the meropenem-vaborbactam combination a valuable agent in managing infections due to KPC-producing carbapenem-resistant *Enterobacteriaceae*.

## Abstract (precis)

Resistance to ceftazidime-avibactam due to mutations in KPC genes has been reported both *in vitro* and in clinical settings. The most frequently reported mutation leads to the amino acid substitution D179Y in the Ω loop of the enzyme. Bacterial cells that carry mutant KPC acquire a higher level of ceftazidime resistance, become more sensitive to other cephalosporins, and almost completely lose resistance to carbapenems. In this study, we demonstrated that two substitutions in KPC-2, D179Y and L169P, reduce the ability of avibactam to enhance the activity of ceftazidime, cefepime, or piperacillin against isogenic efflux-deficient strains of Pseudomonas aeruginosa, 8- to 32-fold and 4- to 16-fold for the D179Y and L169P variants, respectively, depending on the antibiotic.

## INTRODUCTION

The most common mechanism of resistance to β-lactam antibiotics in Gram-negative bacteria is production of β-lactamase enzymes capable of cleaving the β-lactam ring, resulting in a complete loss of activity. Inhibition of β-lactamase activity with small-molecule inhibitors (BLIs) has been a broadly recognized strategy to prevent β-lactam cleavage and restore their potency (1, 2). A notable BLI is avibactam, which is a potent inhibitor of numerous serine (class A, class C, and some class D) enzymes, including KPC carbapenemases (3). It was approved by the FDA in 2015 in combination with ceftazidime (4). An increasing number of reports describe the successful use of ceftazidime-avibactam to treat infections caused by KPC-producing carbapenem-resistant *Enterobacteriaceae* (CRE) (5–9).

Our own efforts led to the discovery of a structurally and mechanistically different BLI, vaborbactam, a cyclic boronate with activity against class A and class C β-lactamases (10). Similar to avibactam, vaborbactam is a potent inhibitor of KPC enzymes (11, 12) and is capable of enhancing the activity of meropenem *in vitro* and in mouse infection models against KPC-producing *Enterobacterales* (13, 14). In 2017, vaborbactam was approved by the FDA in combination with meropenem (15). Its utility to treat infections due to KPC-producing CRE has been demonstrated in a multinational, open-label, randomized clinical trial (16) and in a recently conducted prospective, observational study of patients with CRE infections (17).

An apparent difference between ceftazidime-avibactam and meropenem-vaborbactam is their relative abilities to select for mutations in a target KPC gene. *In vitro* multistep resistance development studies with the meropenem-vaborbactam combination failed to generate any target mutations in KPC genes harbored by various clinical strains (18). No mutations in KPC genes have been reported to date for patients treated with meropenem-vaborbactam. Decreased susceptibility to meropenem-vaborbactam appears to be due to a combination of various mechanisms affecting intracellular accumulation of either meropenem or vaborbactam (porin mutations and increased efflux) (18, 19).

In contrast, *in vitro* single-step resistance development studies using ceftazidime-avibactam as a selective agent have identified several mutations in the *bla*_KPC-3_ gene that conferred resistance to this combination (20). One of these mutations, D179Y, has been also detected worldwide in KPC-2- and KPC-3-producing clinical isolates of *Enterobacteriaceae* recovered from patients after treatment with the ceftazidime-avibactam combination (21–25). Importantly, this mutation concurrently resulted in restoration of susceptibility to carbapenems (24, 26, 27). Not surprisingly, strains containing KPC with the D179Y mutation are also susceptible to meropenem-vaborbactam (19). A recent report documented that treatment with meropenem-vaborbactam resulted in resolution of an infection due to KPC-producing Klebsiella pneumoniae with treatment-emergent ceftazidime-avibactam resistance (28).

It was proposed that ceftazidime-avibactam resistance conferred by the D179 substitutions can be due to stabilizing interactions (e.g., hydrogen bonds) of ceftazidime within the active site of variant β-lactamases that prevent avibactam from binding to and inhibiting the enzyme (29, 30). However, another recent publication demonstrated a significant effect of the D179Y substitution in KPC-2 on the efficiency of avibactam acylation of the enzyme (70,000-fold decrease in the inactivation constant *k*_2_/*K* value) (31).

L169P is another mutation, located close to D179Y in the Ω-loop region of KPC-2, that is associated with ceftazidime-avibactam resistance; it has also been recovered from a patient treated with ceftazidime-avibactam (deposited in GenBank as KPC-35) (32, 33). Similar to the D179Y mutation, it converts clinical isolates to a carbapenem-susceptible phenotype. Currently, no biochemical studies have been published on the mechanism of resistance caused by this mutation.

We initiated a series of studies focusing on the role of partner antibiotic and BLI in selecting for target-based resistance to the combination agent. In this study, we evaluated the effects of the D179Y and L169P mutations on the potency of vaborbactam and avibactam to enhance the activity of various antibiotics in isogenic strains expressing KPC enzymes. Additionally, the effects of these mutations on interaction with substrates and inhibitors were studied at the biochemical level using purified wild-type (WT) and mutant proteins.

## RESULTS AND DISCUSSION

### Effects of amino acid substitutions in KPC-2 on MICs of various antibiotics.

The effects of KPC mutations on resistance to various antibiotics were investigated. For these studies, pUCP24 plasmids carrying wild-type and mutant *bla*_KPC-2_ genes as well as the pUCP24 vector were transformed into PAM1154, an efflux-deficient strain of Pseudomonas aeruginosa. In this host, the effect of β-lactamases on β-lactam MIC is amplified due to the slowed uptake of β-lactams across the low-permeability outer membrane without interference by efflux of either β-lactams or BLIs. Consequently, this host allows detection of β-lactamase activity (as an MIC increase) of low-catalytic-efficiency enzymes that rely heavily on the low permeability of the outer membrane. Using the strain that lacks efflux pumps ensures no interference from efflux in interpreting results. We next evaluated the steady-state protein expression levels in bacterial cells. A Western blotting experiment with whole-cell lysates using anti-KPC-2 antibodies showed no difference in protein expression levels in P. aeruginosa PAM1154 cells expressing both mutant proteins versus wild-type KPC-2 (see Fig. S1 in the supplemental material), suggesting no effect of mutations on protein stability. Previously, various amino acid substitutions at position 179 of KPC-2 were shown to broadly reduce protein expression levels with the D179Y mutant, demonstrating a severalfold decrease compared to the result with wild-type protein (29). The observed discrepancy with our results could be attributed to the difference in either the expression vector or host bacteria.

MIC studies demonstrated that both mutations resulted in a 64-fold reduction of aztreonam and meropenem MICs: from 128 to 2 μg/ml and from 64 to 1 μg/ml for aztreonam and meropenem, respectively. Of note, the MIC of the vector-alone strain for these antibiotics was 0.125 μg/ml, indicating that the mutant KPC still afforded a ca. 8- to 16-fold increase in aztreonam and meropenem MICs (Table 1). Cefepime MICs of the strains that carried mutant KPCs were reduced 4-fold, from 256 to 64 μg/ml, still affording a 512-fold increase in MIC compared to that with the vector-only strain. Piperacillin MICs were reduced 4-fold and 8-fold for the strains with D179Y and L169P mutatations, respectively, from 128 to 32 μg/ml and 16 μg/ml, resulting in a 256- to 512-fold difference in MIC between the strains that carried KPC mutations versus the vector-alone cells. In contrast with those of other antibiotics, ceftazidime MICs were increased by both mutations: 16- and 8-fold increases for the D179Y and L169P mutations, respectively. In general, our MIC results for D179Y are in good agreement with published data for multiple D179 substitutions reported for the KPC-2 enzyme: increased ceftazidime MICs, moderate decrease of MICs of other cephalosporins, and a significant decrease in resistance to monobactams and carbapenems (22, 26, 29, 31, 32). Somewhat higher MICs of aztreonam and meropenem for D179Y reported in our study are most probably due to the host strain, P. aeruginosa as opposed to a more routinely used Escherichia coli, which has a more permeable outer membrane with a consequently higher rate of β-lactam uptake. Avibactam inhibited growth of all the strains with a MIC of 128 μg/ml. The MIC for vaborbactam was >256 μg/ml.

**TABLE 1:** MICs for the P. aeruginosa PAM1154 carrying plasmids with wild-type KPC-2 or the corresponding mutant proteins^*a*^

Strain	Plasmid	MIC (μg/ml) of drug					
		Aztreonam	Meropenem	Ceftazidime	Cefepime	Piperacillin	Avibactam
PAM4175	pUCP24	0.125	0.125	0.25	0.125	0.06	128
PAM4135	pUCP24-KPC-2	128	64	32	256	128	128
PAM4639	pUCP24-KPC-2::D179Y	2	1	512	64	32	128
PAM4751	pUCP24-KPC-2::L169P	2	1	256	64	16	128

a Vaborbactam MICs are >256 μg/ml for all strains.

### Effects of amino acid substitutions in KPC-2 on the potency of avibactam and vaborbactam to enhance the activities of various antibiotics.

The effects of mutations on BLI potency were investigated next. BLI potency was defined as PV_50_ (PV, potentiation value) of antibiotic potentiation. PV_50_ is the concentration of a BLI that is required to reduce the antibiotic MIC to the middle of the MIC range where the highest MIC is the MIC for a KPC-producing strain with no inhibitor added and the lowest MIC is the MIC for the vector-alone strain, which corresponds to complete inhibition of KPC. As a given MIC is directly related to the β-lactamase activity, PV_50_ might be considered the concentration of a BLI that is required to achieve half-effect of inhibition of β-lactamase activity (in whole cells) to hydrolyze an antibiotic of interest. The advantage of using PV_50_ as a measure of BLI potency is that it does not depend on antibiotic MIC. However, the accurate determination of PV_50_ requires the MIC range to be relatively wide. Based on these considerations, ceftazidime, cefepime, and piperacillin were selected for checkerboard experiments.

Both avibactam and vaborbactam caused a dose-dependent decrease of ceftazidime, cefepime, and piperacillin MICs in P. aeruginosa PAM1154 expressing wild-type KPC-2 and its mutants (Table S1). Calculated PV_50_s are presented in Table 2.

**TABLE 2:** MICs of ceftazidime, cefepime, and piperacillin alone or in combination with BLIs for P. aeruginosa PAM1154 containing plasmids with wild-type KPC-2 or the corresponding mutant proteins

Strain	KPC variant	Antibiotic	Antibiotic MIC (μg/ml) alone or with BLI at clinically used concentrations			PV_50_ (μg/ml)^*a*^	
			No BLI	With avibactam at 4 μg/ml	With vaborbactam at 8 μg/ml	Avibactam	Vaborbactam
PAM4175	pUCP24 (vector)	Ceftazidime	0.25	0.25	0.25		
PAM4135	KPC-2	Ceftazidime	16	0.25	0.25	0.125	0.5
PAM4639	KPC-2::D179Y	Ceftazidime	256	8	1	4	1
PAM4751	KPC-2::L169P	Ceftazidime	128	2	0.5	2	1
							
PAM4175	pUCP24 (vector)	Cefepime	0.125	0.125	0.125		
PAM4135	KPC-2	Cefepime	64	0.125	0.125	0.25	0.5
PAM4639	KPC-2::D179Y	Cefepime	32	2	0.25	4	1
PAM4751	KPC-2::L169P	Cefepime	32	0.5	0.125	1	1
							
PAM4175	pUCP24 (vector)	Piperacillin	0.125	0.125	0.125		
PAM4135	KPC-2	Piperacillin	128	0.5	0.25	0.25	1
PAM4639	KPC-2::D179Y	Piperacillin	32	1	0.25	1	1
PAM4751	KPC-2::L169P	Piperacillin	16	0.5	0.25	2	1

a PV_50_ is a minimal concentration of a BLI that is required to reduce the antibiotic MIC to the middle of the MIC range (E_50_) where the highest MIC is the MIC for a KPC-producing strain with no inhibitor added and the lowest MIC is the MIC for vector-only strain, corresponding to the complete inhibition of KPC. E_50_ is calculated as the square root of the product of the antibiotic MICs for the KPC-producing and vector-only strains.

The D179Y substitution in KPC-2 appeared to increase the avibactam PV_50_s 4-, 16-, and 32-fold for piperacillin, cefepime, and ceftazidime, respectively, compared to the values with wild-type KPC-2. The L169P mutation also appeared to decrease avibactam potency, albeit to a somewhat lesser degree: 4- and 8-fold increases in PV_50_ for cefepime or piperacillin and ceftazidime, respectively. Of note, a decreased avibactam potency to reduce the MICs of the three tested antibiotics against the strains producing mutant proteins compared to the wild-type KPC-2 was observed, irrespective of the effect of mutations on antibiotics: reduction of cefepime and piperacillin MIC and an increase in ceftazidime MIC. This result is indicative of a possible direct effect of mutations on avibactam affinity for the KPC β-lactamase. A recent study reported the impact of the D179Y substitution on activity of another BLI, clavulanic acid, which became more potent than avibactam in potentiating ceftazidime against a strain of E. coli with a cloned KPC-2::D179Y variant while being much less potent than avibactam against the wild-type protein (31). However, since clavulanic acid is efficiently hydrolyzed by KPC (34), it is conceivable that a KPC variant that carries a D179Y substitution can lose the ability to inactivate clavulanic acid and as a result become more susceptible to inhibition.

Potentiation experiments with vaborbactam demonstrated that D179Y and L169P substitutions appear to affect PV_50_ of vaborbactam to a much lesser degree than for avibactam. None of the mutations decreased the potency of vaborbactam to enhance the activity of ceftazidime, cefepime, or piperacillin more than 2-fold (Table 2). While avibactam appeared to be 2- to 4-fold more potent than vaborbactam to potentiate antibiotics against the strain producing the wild-type KPC-2 (avibactam PV_50_s of 0.125 μg/ml, 0.25 μg/ml, and 0.25 μg/ml versus vaborbactam PV_50_s of 0.5, 0.5, and 1 μg/ml for ceftazidime, cefepime, and piperacillin, respectively), it became 4- and 2-fold less potent as a potentiator against the strain producing the D179Y and L169P variants, respectively. These results indicate that the D179Y and L169P substitutions in the KPC-2 β-lactamase had a lesser effect on interaction with vaborbactam. Importantly, when antibiotic MICs against the strains producing KPC mutants were determined with BLIs at clinically relevant concentrations (4 μg/ml and 8 μg/ml for avibactam and vaborbactam, respectively), they were consistently lower for vaborbactam combinations than for avibactam combinations. The highest difference, 8-fold, was for ceftazidime versus the strain producing D179Y variant: 8 μg/ml and 1 μg/ml for ceftazidime-avibactam and ceftazidime-vaborbactam, respectively.

The detection of the apparent impact of KPC mutations on interaction with avibactam (and to a much lesser degree with vaborbactam) became possible by generating complete concentration response curves of MIC of antibiotics versus BLI concentration for wild-type and mutant strains. Comparing MICs of antibiotics against the wild type and its mutants at a single inhibitor concentration might not allow discrimination between the effect of a mutation solely on antibiotic MIC (as was proposed based on earlier microbiological studies [20]) versus an additional possible direct effect on interaction with a BLI.

While the effect of mutations on BLI affinity is obviously an important factor that might contribute to resistance to a β-lactam–BLI combination, the effect on antibiotic MIC is another factor potentially contributing to resistance. As an example, the MICs of ceftazidime and cefepime against the strain producing the D179Y variant are 256 μg/ml and 32 μg/ml, respectively, and the PV_50_ for avibactam to potentiate ceftazidime or cefepime against this strain was found to be 4 μg/ml. At this concentration, the ceftazidime MIC is 8 μg/ml and the cefepime MIC is 4-fold lower, or 2 μg/ml (Table 2). Of note, the group who first selected ceftazidime-avibactam-resistant mutants of KPC failed to get such selection in earlier studies with ceftaroline-avibactam (35). It will be very interesting to investigate whether it is purely due to a decrease in a ceftaroline MIC (35). Irrespectively, the above result underscores the importance of a partner antibiotic when considering a combination with a β-lactamase inhibitor.

In conclusion, our microbiological studies point to both the reduced affinity to avibactam (increased PV_50_ for avibactam) and the specific role of ceftazidime in ceftazidime-avibactam resistance conferred by the D179Y and L169P substitutions. These data also indicate that the potency of vaborbactam is affected by KPC mutations to a lesser degree than that of avibactam.

### Effects of amino acid substitutions in KPC-2 on β-lactamase activity.

Biochemical studies with purified wild-type KPC-2 and mutant proteins were carried out in an attempt to verify our microbiological results and potentially gain more mechanistic understanding on a biochemical level. First, we used a reporter substrate, nitrocefin, to determine kinetic parameters of KPC-2 variants. Purified D179Y and L169P proteins demonstrated nitrocefin *K_m_* values of 26 ± 12 μM and 40 ± 10 μM, which are similar to those of the wild-type KPC-2 (Table 3). In contrast, the D179Y and L169P mutants exhibited approximately 6,000- and 300-fold reductions of nitrocefin *k*_cat_ values, respectively. Of note, a similar reduction in *k*_cat_ values of hydrolysis of another chromogenic cephalosporin, CENTA, by the D179Y variant was also reported in a recent study (31). This drop of hydrolytic activity for the mutant enzymes supports our microbiological observations that the strains expressing mutant KPC-2 proteins lost resistance to most antibiotics except ceftazidime and some cephalosporins. Next, we attempted to determine the kinetic parameters of ceftazidime hydrolysis for all three enzymes. Michaelis-Menten plots of reaction velocity versus substrate concentration are presented in Fig. S2. Wild-type KPC-2 demonstrated no sign of saturation of reaction velocity with an increase of substrate concentration up to 2,000 μM, indicating that the *K_m_* value is higher than that number and preventing separate calculations of *K_m_* and *k*_cat_ values. This finding is in good agreement with the previously reported data (36, 37). In contrast, the rate of ceftazidime hydrolysis for the D179Y mutant remained constant at a wide range of substrate concentrations, suggesting that the *K_m_* value is very low (below the detection limit); thus, this type of kinetic behavior allows only calculation of the *k*_cat_ value (Table 3; Fig. S2). Accordingly, for wild-type KPC-2 and the D179Y mutant, we used a different method that allows calculation of the *k*_cat_/*K_m_* catalytic ratio by analyzing complete ceftazidime cleavage profiles. For the KPC-2 wild type, the *k*_cat_/*K_m_* value was 0.00087 ± 0.00011 μM^−1^ s^−1^, while for D179Y, this value was 10-fold higher, 0.0088 ± 0.0009 μM^−1^ s^−1^.

**TABLE 3:** Kinetic parameters of nitrocefin and ceftazidime hydrolysis by KPC-2 and mutant proteins

Enzyme	Nitrocefin			Ceftazidime		
	*K_m_*, μM	*k*_cat_, s^−1^	*k*_cat_/*K_m_*, s^−1^ μM^−1^	*K_m_*, μM	*k*_cat_, s^−1^	*k*_cat_/*K_m_*, s^−1^ μM^−1^
KPC-2	36 ± 5	132 ± 13	3.7 ± 0.2	>2,000	>2.6	0.00087 ± 0.00011^*a*^
KPC-2 D179Y	26 ± 12	0.023 ± 0.002	0.0010 ± 0.0005	<5	0.0088 ± 0.0003	0.0088 ± 0.0009^*a*^
KPC-2 L169P	40 ± 10	0.47 ± 0.04	0.012 ± 0.002	27 ± 6	0.093 ± 0.009	0.0036 ± 0.0009

a This *k*_cat_/*K_m_* value was calculated by method described in reference 44.

Unlike wild-type KPC-2 and the D179Y mutant, the L169P mutant demonstrated a typical Michaelis-Menten plot, with a *K_m_* value of 27 ± 6 μM and *k*_cat_ of 0.093 ± 0.009 s^−1^ (Table 3; Fig. S2); hence, the *k*_cat_/*K_m_* value for the L169P mutant was obtained from separate *K_m_* and *k*_cat_ numbers and was 0.0036 ± 0.0009 μM^−1^ s^−1^.

The observed increase in *k*_cat_/*K_m_* for both mutants may explain the higher ceftazidime MICs observed for the cloned mutant KPC genes (Table 1). The predicted ceftazidime *K_m_* of the D179Y mutant (calculated by division of *k*_cat_ by *k*_cat_/*K_m_*) is around 1 μM, which is more than 2,000-fold lower than that of wild-type KPC-2. Consistent with our findings, a quick burst of ceftazidime hydrolysis has been reported for the KPC-2 D179N mutant when studied using the stop flow kinetic technique; the authors believe that this burst of ceftazidime hydrolysis was due to the tight ceftazidime binding to the mutant protein (29). It is also conceivable that the high increase in affinity to ceftazidime for the D179Y mutant could prevent avibactam from efficiently binding to the enzyme and thus contribute to the resistance mechanism.

### Effects of amino acid substitutions in KPC-2 on inhibition by avibactam and vaborbactam.

We next attempted to determine the effect of mutations on interactions with avibactam and vaborbactam using nitrocefin as a substrate. It has been reported that both BLIs behave as slow tightly binding inhibitors of KPC-2, which is presented by the following kinetic scheme (12, 38).

$$\text{E}+\text{I}\overset{k_{1}}{\underset{k_{-1}}{↔}}\text{EI}\overset{k_{2}}{\underset{k_{-2}}{↔}}\text{EI*},\text{where }K=k_{-1}/k_{1}\text{ and }K_{d}=K\times k_{-2}/(k_{2}+k_{-2})$$

This type of inhibition is manifested by a progressive inactivation phenomenon when studied using the “reporter substrate” method (Fig. S3). Consequently, the affinity of such inhibitors to β-lactamases is quantitatively characterized by the inactivation constant *k*_2_/*K* (*k_on_*) and off-rate constant *k*_−2_, with overall BLI affinity characterized by *K_d_* (dissociation constant) values We have recently reported vaborbactam inhibition parameters for KPC-2 (12), and in this study, we determined the avibactam inhibition parameters (Table 4). Our data are very similar to the previously reported results (38). Avibactam and vaborbactam *K_d_*s for KPC-2 were very similar, 0.014 μM and 0.008 μM, respectively (Table 4). Unexpectedly, the KPC-2 L169P mutant protein produced linear enzyme inactivation profiles with both inhibitors that are typical of “fast on–fast off” boronic BLIs (Fig. S4). This finding precluded calculation of *k*_2_/*K* and *k*_−2_ values for this mutant. Hence, avibactam and vaborbactam steady-state *K_i_* values were determined for the L169P mutant using the method that has been previously utilized for fast on–fast off boronic BLIs with nitrocefin as a substrate (39, 40). *K_i_* values were found to be 0.89 ± 0.03 μM and 0.19 ± 0.02 μM for avibactam and vaborbactam, respectively, or ∼4.5-fold higher for avibactam than for vaborbactam (Table 4).

**TABLE 4:** Kinetic parameters of vaborbactam and avibactam inhibition of NCF hydrolysis by KPC-2 and the L169P mutant

Enzyme	Avibactam				Vaborbactam			
	*k*_2_/*K*, M^−1^ s^−1^	*k*_−2_, s^−1^	*K_d_*, μM	*K_i_*, μM	*k*_2_/*K*, M^−1^ s^−1^	*k*_−2_, s^−1^	*K_d_*, μM	*K_i_*, μM
KPC-2	2.3 ± 0.2 × 10^4^	3.3 ± 0.1 × 10^−4^	0.014 ± 0.001		5.5 ± 0.5 × 10^3^	4.3 ± 0.6 × 10^−5^	0.0078 ± 0.0011	
KPC-2 L169P				0.89 ± 0.03				0.19 ± 0.02

The apparent difference in inhibition kinetics observed for wild-type KPC-2 and its L169P derivative precluded us from directly comparing inhibition constants observed for each inhibitor for wild-type versus mutant KPC-2. However, while the two inhibitors apparently had similar affinities for wild-type KPC-2, the affinity of avibactam for the mutant L169P protein was ∼4.5-fold lower than for vaborbactam (Table 4). This result indicated that the L169P amino acid substitution in KPC-2 affected avibactam more strongly than vaborbactam, which is similar to the results from microbiological experiments (2-fold to 8-fold-stronger effect of the L169P substitution on avibactam PV_50_ versus vaborbactam PV_50_ depending on the antibiotic). The logical conclusion is that the L169P substitution does affect the affinity of avibactam for the KPC-2 β-lactamase, though the exact magnitude of the effect remains to be determined in future experiments. This potential reduced affinity for avibactam can contribute to ceftazidime-avibactam resistance in addition to the previously described increase in ceftazidime hydrolytic activity observed for this mutant.

The results of evaluation of the impact of avibactam and vaborbactam on nitrocefin hydrolysis mediated by the D179Y mutant were unexpected. Avibactam did not inhibit this hydrolysis even when used at the very high concentration of 2,560 μM. Vaborbactam demonstrated some inactivation effect at 2,560 μM, but a rough estimation of the *k*_2_/*K* value resulted in an almost 10,000-fold decrease in inactivation efficiency compared to that of wild-type KPC-2 (Fig. S4). Both avibactam and vaborbactam findings are at odds with our microbiological data that indicate that both inhibitors are capable of enhancing the activities of various antibiotics against the strain producing the D179Y mutation with potency that is only 2-fold or 8- to 32-fold lower for vaborbactam and avibactam, respectively, compared to that observed against the strain producing wild-type KPC-2 β-lactamase (Table 2). We believe that the abnormally high resistance to inhibition observed in the nitrocefin (and possibly, CENTA) hydrolysis assay with the D179Y mutant might be an artifact attributable to the extremely low rate of nitrocefin hydrolysis.

Owing to the ability of the D179Y variant to hydrolyze ceftazidime, we attempted to compare binding affinities of avibactam and vaborbactam for wild-type KPC-2 and the D179Y mutant by measuring the 50% inhibitory concentration (IC_50_) for inhibition of ceftazidime hydrolysis. To account for the different ceftazidime *K_m_* values for these proteins, ceftazidime was used at 100 μM and 10 μM for wild-type KPC-2 and the D179Y mutant, respectively. The results are presented in Table 5. We first compared the potencies of avibactam and vaborbactam against the same KPC variant. For wild-type KPC-2, the IC_50_ of avibactam (0.47 ± 0.02 μM) was ca. 2-fold lower than that of vaborbactam (0.94 ± 0.02 μM), somewhat reminiscent of the 2- to 4-fold-lower PV_50_s for avibactam obtained in microbiological experiments (Table 2). In contrast, for the D179Y mutant protein, the IC_50_ of avibactam (8.9 ± 0.9 μM) was ca. 4.5-fold higher (reduced potency) than that of vaborbactam (1.9 ± 0.3 μM) (Table 5), again somewhat similar to the 4-fold-lower potency (higher PV_50_s) of avibactam than of vaborbactam for the strain producing KPC-2 with the D179Y substitution.

**TABLE 5:** IC_50_s of vaborbactam and avibactam inhibition of ceftazidime hydrolysis by KPC-2 and the D179Y mutant^*a*^

Enzyme	IC_50_, μM	
	Avibactam	Vaborbactam
KPC-2	0.47 ± 0.02	0.94 ± 0.02
KPC-2 D179Y	8.9 ± 0.9	1.9 ± 0.3

a Concentrations of 100 μM and 10 μM ceftazidime were used to determine IC_50_s for KPC-2 and its D179Y mutant, respectively.

We are precluded from establishing whether the D179Y substitution affected the affinity of BLIs for KPC-2, let alone accurately estimating the magnitude of its potential effect, by comparing IC_50_s for the wild-type versus mutant KPC-2 that were determined at different substrate saturation conditions. However, it is possible to compare avibactam and vaborbactam based on the impact caused by the mutation by comparing the changes in IC_50_. The avibactam IC_50_ was increased almost 20-fold, from 0.47 ± 0.02 μM for wild-type KPC-2 to 8.9 ± 0.9 μM for D179Y (Table 5). At the same time, the vaborbactam IC_50_ was increased only 2-fold, from 0.94 ± 0.02 μM to 1.9 ± 0.3 μM, indicating the different impacts of D179Y on two different BLIs. Based on this result, we conclude that the D179Y substitution has a direct effect on the affinity of avibactam and vaborbactam but the effect on avibactam is stronger than that on vaborbactam. As was the case with L169P, the determination of the exact magnitude of the effect on inhibition parameters will probably require a different experimental technique.

### Conclusions.

In conclusion, our microbiological studies pointed both to the specific role of ceftazidime, possibly due to increased efficiency of ceftazidime hydrolysis, and to the reduced affinity to avibactam in ceftazidime-avibactam resistance conferred by the D179Y and L169P substitutions. These studies were in a good agreement with biochemical experiments.

Purified KPC-2 D179Y and L169P enzymes demonstrated a higher catalytic ratio (*k*_cat_/*K_m_*) of ceftazidime hydrolysis than did the wild-type protein, as well as significantly reduced ceftazidime *K_m_* values. It is plausible that the former may explain the increased ceftazidime resistance associated with both mutations while the latter is responsible for the observed reduction of avibactam potency to enhance activity of ceftazidime in microbiological experiments. In addition, the D179Y and L169P substitutions appeared to have direct effects on avibactam binding affinity for KPC-2; this may explain the negative impact of both mutations on potentiation of other antibiotics. These mutations had a lesser effect on both the enzyme inhibition and antibiotic potentiation activity of vaborbactam. In addition, KPC mutations that confer resistance to ceftazidime-avibactam resulted in significantly reduced resistance to meropenem. This makes the meropenem-vaborbactam combination a valuable agent in managing infections due to KPC-producing carbapenem-resistant *Enterobacteriaceae*.

## MATERIALS AND METHODS

### Generation of KPC-2 mutants.

Mutations in the *bla*_KPC-2_ gene cloned in either pUCP24 or pET28a plasmids were introduced using the QuikChange Lightning site-directed mutagenesis kit (Thermo Fisher Scientific, USA).

### Susceptibility testing.

For microbiological studies, wild-type *bla*_KPC-2_ and its mutant variants were cloned into the shuttle vector pUCP24. Resulting plasmids were transformed in the efflux-deficient strain of P. aeruginosa PAM1154 using selection on 15 μg/ml of gentamicin. Using a strain of P. aeruginosa as opposed to E. coli as a host for various cloned genes was based on the following consideration. As well documented elsewhere (41), P. aeruginosa has a low-permeability outer membrane; consequently, the effect of β-lactamases on MIC is amplified in P. aeruginosa compared to that in E. coli due to the slowed uptake of β-lactams. Thus, this host is appropriate to detect β-lactamase activity (as an MIC increase) of low-catalytic-efficiency enzymes that rely heavily on low permeability of the outer membrane. PAM1154 lacks major efflux pumps, so efflux does not interfere with microbiological potency of either β-lactams or β-lactamase inhibitors. MICs were determined using the Clinical and Laboratory Standards Institute (CLSI) broth microdilution method as described in CLSI document M07-A11 (42). Potentiation of antibiotic activity by various BLIs in bacterial strains carrying wild-type (WT) and mutant KPC-2 genes was performed using standard checkerboard methodology (43). BLI potency was defined as PV_50_ (PV, potentiation value). PV_50_ is the minimal concentration of a BLI that is required to reduce the antibiotic MIC to the middle of the MIC range (E_50_) where the highest MIC is the MIC for a KPC-producing strain with no inhibitor added and the lowest MIC is the MIC for the vector-only strain, corresponding to the complete inhibition of KPC. E_50_ is calculated as the square root of the product of the antibiotic MICs for the KPC-producing and the vector-only strains.

The results of checkerboard experiments were highly reproducible.

### Evaluation of KPC-2 mutant protein expression level in P. aeruginosa strain PAM1154.

Bacterial cells carrying plasmids expressing the KPC-2 WT and mutants were grown in liquid media to an optical density at 600 nm (OD_600_) of 0.7 to 0.9 and diluted to a final OD_600_ of 0.5. A total of 500 μl of cell culture was spun down and the resulting pellet was resuspended in 500 μl of gel loading buffer. Twenty microliters of cell lysate was loaded onto 8 to 16% SDS-PAGE gels. After transfer, the membrane was probed with custom-produced rat polyclonal anti-KPC-2 antibodies and subsequently treated with secondary goat anti-rat horseradish peroxidase (HRP)-conjugated antibodies. Anti-RNA polymerase β-subunit monoclonal antibodies (Abcam, Burlingame, CA; ab12087) were used as a loading control.

### Purification of wild-type KPC-2 and D179Y mutant proteins.

KPC-2 gene coding sequence was cloned into a pET28a vector that produced the construct with periplasmic KPC-2 secretion and a 6×His tag on its C terminus. The recombinant plasmids were transformed into the BL21(DE3) pLys strain. Protein expression was induced with 0.2 mM isopropyl-β-d-thiogalactopyranoside (IPTG) for 3 h at 37°C. The cell pellet was lysed in ice-cold 50 mM Tris HCl (pH 8.0)–500 mM sucrose–1 mM EDTA with six cycles of 15-s vortexing and 5 min of incubation on ice. After centrifugation, the supernatant was adjusted with MgCl_2_ and imidazole to 5 mM and 4 mM, respectively. The lysate was loaded onto a 1-ml column with HisPur cobalt resin (Thermo Fisher Scientific, USA) preequilibrated with 50 mM sodium phosphate (pH 7.4)–300 mM NaCl–4 mM imidazole buffer. The column was washed with 40 ml of the same buffer, and consequently, the His tag protein was eluted with a linear gradient of 4 mM to 70 mM imidazole in 50 mM sodium phosphate (pH 7.4)–300 mM NaCl buffer. All fractions were analyzed by 8 to 16% SDS-PAGE. Fractions containing target protein were pooled, concentrated, and dialyzed against 50 mM sodium phosphate (pH 7.0).

### Purification of KPC-2 L169P mutant protein.

Protein expression was induced with 0.2 mM IPTG overnight at 18°C. The cell pellet was lysed in ice-cold 50 mM sodium phosphate (pH 7.4)–300 mM NaCl–4 mM imidazole with seven cycles of 1-min sonication on ice. After centrifugation, the lysate was loaded onto a 1-ml column with HisPur cobalt resin preequilibrated with 50 mM sodium phosphate (pH 7.4)–300 mM NaCl–4 mM imidazole buffer. The column was washed with 40 ml of the same buffer, and consequently, the His tag protein was eluted with a linear gradient of 4 mM to 70 mM imidazole in 50 mM sodium phosphate (pH 7.4)–300 mM NaCl buffer. All fractions were analyzed by 8 to 16% SDS-PAGE. Fractions containing target protein were pooled, concentrated, and dialyzed against 50 mM sodium phosphate (pH 7.0).

### Determination of *K_m_* and *k*_cat_ values for nitrocefin and ceftazidime cleavage by KPC-2 WT and mutant proteins.

Enzymes were mixed with various concentrations of nitrocefin in 50 mM sodium phosphate (pH 7.0)–0.1 mg/ml of bovine serum albumin (buffer A), and substrate cleavage was monitored at 490 nm every 10 s for 10 min at 37°C on a SpectraMax plate reader (Molecular Devices, San Jose, CA). Initial rates of nitrocefin cleavage were calculated and used to obtain *K_m_* and *k*_cat_ values with Prism software (GraphPad, San Diego, CA). For ceftazidime kinetic parameter calculation, enzymes were mixed with various concentrations of substrate in buffer A and transferred in either a 1-mm- or 10-mm-light-path quartz cuvette, and substrate cleavage was monitored at 260 nm every 30 s for 10 min at room temperature. Initial rates of ceftazidime cleavage were calculated and used to obtain *K_m_* and *k*_cat_ values with Prism software.

### Determination of *k*_cat_/*K_m_* ratio for ceftazidime cleavage by purified enzymes.

Wild-type KPC-2 enzyme was mixed with 250 μM ceftazidime in buffer A, and the reaction mixture was transferred to a 1-mm-light-path quartz cuvette. For D179Y and L169P mutants, enzyme was mixed with 2.5 μM ceftazidime in buffer A and the reaction mixture was transferred to a 10-mm-light-path quartz cuvette. Substrate cleavage was monitored at 260 nm every 30 s at room temperature using a SpectraMax plate reader. The reaction was monitored until OD_260_ values reached a plateau. Resulting OD_260_ versus time reaction profiles were fitted to the following equation using Prism software: *A_t_* = *A*_∞_ + (*A*_0_ − *A*_∞_) × *e*^−k×t^, where *A_t_* is absorbance at time *t*, *A*_0_ is initial absorbance, and *A*_∞_ is final absorbance. In this equation, *k* = *k*_cat_/*K_m_* × [E], which allows calculatation of *k*_cat_/*K_m_* knowing the enzyme concentration (44).

### Determination of avibactam *k*_2_/*K* inactivation constant for KPC-2 enzyme.

Inactivation kinetic parameters were determined by reporter substrate method for the slow tight binding inhibitor kinetic scheme (45). Protein was quickly mixed with 100 μM nitrocefin and various concentrations of BLI in reaction buffer, and absorbance at 490 nm was measured immediately every 2 s for 600 s on a SpectraMax plate reader (Molecular Devices, San Jose, CA) at 37°C. Resulting progression curves of OD_490_ versus time at various BLI concentrations were imported into Prism software (GraphPad, San Diego, CA), and pseudo-first-order rate constants (*k*_obs_) were calculated using the following equation:

$$P=V_{\text{s}}\times (1-e^{-k_{\text{obs}}}{{}^{\times }}^{t})\text{/}k_{\text{obs}}$$

where *V*_s_ is enzyme nitrocefin cleavage rate in the absence of BLI. *k*_obs_ values calculated at various vaborbactam concentrations were fitted in the following equation:

$$k_{\text{obs}}=k_{-2}+k_{2}/K\times \left[\text{I}\right]/\left\{1+\left[\text{NCF}\right]/K_{m}\left(\text{NCF}\right)\right\}$$

where *k*_2_/*K* is the inactivation constant, [I] is inhibitor concentration, [NCF] is nitrocefin concentration, and *K_m_*(NCF) is the Michaelis constant of nitrocefin for KPC-2.

### Determination of *k*_−2_ rates of KPC-2 enzyme activity recovery after inhibition by avibactam.

Purified KPC-2 at a 1 μM concentration in buffer A was mixed with BLIs at an 8-fold-higher concentration than its stoichiometry ratio (determined in preliminary stoichiometry experiments). After 30 min of incubation at 37°C, the reaction mixture was diluted 10,000-fold in buffer A and 100 μl of diluted enzyme was mixed with 100 μl of 400 μM nitrocefin in reaction buffer. Absorbance at 490 nm was recorded every minute for 4 h at 37°C. Resulting reaction profiles were fitted into the following equation using GraphPad Prism software to obtain *k*_−2_ values:

$$P=V_{\text{s}}\times t+(V_{\text{o}}-V_{\text{s}})\times (1-e^{-k_{-2}}{{}^{\times }}^{t})/k_{-2}$$

where *V*_s_ is uninhibited enzyme velocity, measured in the reaction with enzyme and no inhibitor, and *V*_o_ is completely inhibited enzyme velocity, measured in the reaction with no enzyme and nitrocefin only.

### Determination of *K_i_* values of β-lactamase inhibition by BLIs with nitrocefin as a substrate.

Enzymes were mixed with BLIs at concentrations varying from 160 to 0.0027 μM in buffer A and incubated for 10 min at 37°C, and NCF substrate was subsequently added. Substrate cleavage profiles were recorded at 490 nm at 37°C every 10 s for 10 min. *K_i_* values were calculated by method of Waley (46). The reaction profiles with and without inhibitor are compared, and several values of the time difference (*t* − *t_c_*, where *t* and *t_c_* are times to reach given substrate concentration in the reaction with and without inhibitor, respectively) for equal values of substrate concentration *s* are obtained. Then the time difference is plotted against ln(*s*_0_/*s*) (*s*_0_ is initial substrate concentration) and the slope is measured. *K_i_* is calculated using the following equation:

$$K_{i\text{app}}=[\text{I}]\times \frac{K_{m}}{V_{\text{max}}}\times \frac{1}{\text{slope}}$$

where *K_i_*_app_ is apparent *K_i_*, [I] is inhibitor concentration, and *K_m_* and *V*_max_ are Michaelis-Menten constants of NCF for KPC-2 and mutant proteins.

### Determination of IC_50_s of inhibition of KPC-2 variants by BLIs with ceftazidime as a substrate.

Enzymes were mixed with BLIs at concentrations varying from 160 to 0.0027 μM in buffer A and incubated for 10 min, and 100 μM (for wild-type KPC-2) or 10 μM (for the D179Y mutant) CFTZ was subsequently added. Substrate cleavage profiles were recorded at 260 nm every 10 s for 10 min. Initial rates of reaction were calculated and exported to Prism software to calculate IC_50_s using the “dose-response – inhibition, variable slope (four parameters)” equation.

### Statistical analysis.

All kinetic results are presented as averages ± standard deviations from a minimum of three replicates.

## Supplementary Material

Supplemental file 1
