# Supplementary material for: Potency of Vaborbactam Is Less Affected than That of Avibactam in Strains Producing KPC-2 Mutations That Confer Resistance to Ceftazidime-Avibactam
Source: Antimicrob Agents Chemother. 2020 Mar 24;64(4):e01936-19. doi: 10.1128/AAC.01936-19 (PMC7179312; doi:10.1128/AAC.01936-19)
Supplement: Supplemental file 1 [file AAC.01936-19-s0001.pdf]

Supplementary Tables

Table 1S MIC values (µg/ml) of ceftazidime, cefepime and piperacillin alone or in combination with BLIs at various concentrations (µg/ml) for the *P. aeruginosa* PAM1154 containing plasmids with the wild type KPC-2 or the corresponding mutant proteins: complete concentration response

				Beta-lactam MIC (µg/ml) in the presence of various concentrations of BLIs (µg/ml)																	
Strain	Plasmid	BLI	Beta-lactam	0	0.008	0.016	0.031	0.06	0.125	0.25	0.5	1	2	4	8	16	32	64	128	E_{50}	PV_{50}
PAM4175	pUCP24	Avibactam	Ceftazidime	0.25	0.25	0.25	0.25	0.25	0.25	0.25	0.25	0.25	0.25	0.25	0.25	0.25	0.25	0.125	NG		
PAM4135	pUCP24-KPC-2	Avibactam	Ceftazidime	16	16	8	8	8	2	1	1	0.25	0.25	0.25	0.25	0.25	0.25	0.125	NG	2	0.125
PAM4639	pUCP24-KPC-2 D179Y	Avibactam	Ceftazidime	256	256	256	256	256	256	128	64	32	16	8	8	2	1	0.25	NG	8	4
PAM4751	pUCP24-KPC-2 L169P	Avibactam	Ceftazidime	128	128	128	128	128	64	32	16	8	4	2	1	0.5	0.5	0.25	NG	5.7	2
PAM4175	pUCP24	Vaborbactam	Ceftazidime	0.25	0.25	0.25	0.25	0.25	0.25	0.25	0.25	0.25	0.25	0.25	0.25	0.25	0.25	0.25	0.25		
PAM4135	pUCP24-KPC-2	Vaborbactam	Ceftazidime	16	16	16	16	16	8	4	1	0.25	0.25	0.25	0.25	0.25	0.25	0.25	0.25	2	0.5
PAM4639	pUCP24-KPC-2 D179Y	Vaborbactam	Ceftazidime	256	256	256	256	256	256	128	32	8	4	1	1	1	0.5	0.5	0.5	8	1
PAM4751	pUCP24-KPC-2 L169P	Vaborbactam	Ceftazidime	128	128	128	128	128	128	64	8	2	1	0.5	0.5	0.25	0.25	0.25	0.25	5.7	1
PAM4175	pUCP24	Avibactam	Cefepime	0.125	0.125	0.125	0.125	0.125	0.125	0.125	0.125	0.125	0.125	0.125	0.125	0.125	0.125	0.06	NG		
PAM4135	pUCP24-KPC-2	Avibactam	Cefepime	64	64	64	64	32	4	1	0.5	0.25	0.25	0.125	0.125	0.125	0.06	0.06	NG	2.8	0.25
PAM4639	pUCP24-KPC-2 D179Y	Avibactam	Cefepime	32	32	32	32	32	32	16	16	4	4	2	1	0.5	0.25	0.06	NG	2.0	4
PAM4751	pUCP24-KPC-2 L169P	Avibactam	Cefepime	32	32	32	32	32	16	8	4	2	1	0.5	0.25	0.125	0.125	0.06	NG	2.0	1
PAM4175	pUCP24	Vaborbactam	Cefepime	0.125	0.125	0.125	0.125	0.125	0.125	0.125	0.125	0.125	0.125	0.125	0.125	0.125	0.125	0.125	0.125		
PAM4135	pUCP24-KPC-2	Vaborbactam	Cefepime	64	64	64	64	64	32	16	2	0.25	0.125	0.125	0.125	0.125	0.125	0.125	0.125	2.8	0.5
PAM4639	pUCP24-KPC-2 D179Y	Vaborbactam	Cefepime	32	32	32	32	32	32	16	8	1	0.5	0.25	0.25	0.125	0.125	0.125	0.125	2.0	1
PAM4751	pUCP24-KPC-2 L169P	Vaborbactam	Cefepime	32	32	32	32	32	32	16	4	0.5	0.25	0.125	0.125	0.125	0.125	0.125	0.125	2.0	1
PAM4175	pUCP24	Avibactam	Piperacillin	0.125	0.125	0.125	0.125	0.125	0.125	0.125	0.125	0.06	0.06	0.06	0.06	0.06	0.06	0.03	NG		
PAM4135	pUCP24-KPC-2	Avibactam	Piperacillin	128	128	128	128	64	16	4	2	1	0.5	0.5	0.25	0.125	0.06	0.03	NG	4.0	0.25
PAM4639	pUCP24-KPC-2 D179Y	Avibactam	Piperacillin	32	32	32	32	32	16	16	8	2	2	1	0.5	0.25	0.125	0.125	NG	2.0	1
PAM4751	pUCP24-KPC-2 L169P	Avibactam	Piperacillin	16	16	16	16	16	8	8	4	2	1	0.5	0.25	0.125	0.125	0.03	NG	1.4	2
PAM4175	pUCP24	Vaborbactam	Piperacillin	0.125	0.125	0.125	0.125	0.125	0.125	0.125	0.125	0.125	0.125	0.125	0.125	0.125	0.06	0.06	0.06		
PAM4135	pUCP24-KPC-2	Vaborbactam	Piperacillin	128	128	128	128	128	64	64	8	1	0.5	0.25	0.125	0.125	0.125	0.125	0.063	4.0	1
PAM4639	pUCP24-KPC-2 D179Y	Vaborbactam	Piperacillin	32	32	32	32	32	16	16	4	1	0.5	0.25	0.25	0.125	0.125	0.125	0.063	2.0	1
PAM4751	pUCP24-KPC-2 L169P	Vaborbactam	Piperacillin	16	16	16	16	16	16	8	4	1	0.5	0.25	0.125	0.125	0.125	0.125	0.063	1.4	1

NG, no growth

PV_{50}, a concentration of the BLI to achieve 50% of antibiotic potentiation effect or a concentration of the BLI to reduce antibiotic MIC to or below the middle point (E_{50}) of the MIC range between the MIC of the beta-lactamase producing strain and the MIC of the vector only strain. The middle point is calculated as the square root of the product of the antibiotic MIC values for the beta-lactamase-producing and the vector only strain. MIC values of various antibiotics at a concentration of BLIs at PV_{50} are marked with yellow color for avibactam and green color for vaborbactam.

Supplementary Figures

Figure S1. Western blot analysis of expression level of KPC-2 wt and mutant proteins in *P. aeruginosa* PAM1154 cells.

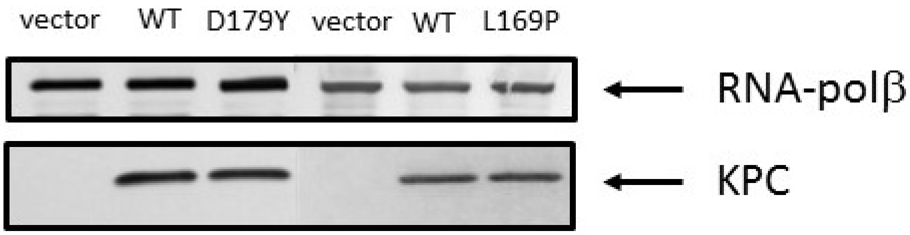

Whole cell protein lysates were separated on 8-16% SDS-PAGE and transferred to PVDF membrane. After transfer membrane was probed with rat polyclonal anti-KPC-2 antibodies or anti-RNA polymerase β-subunit monoclonal antibodies

Figure S2. Rates of ceftazidime hydrolysis plotted vs substrate concentration for KPC-2 wild type and mutant proteins.

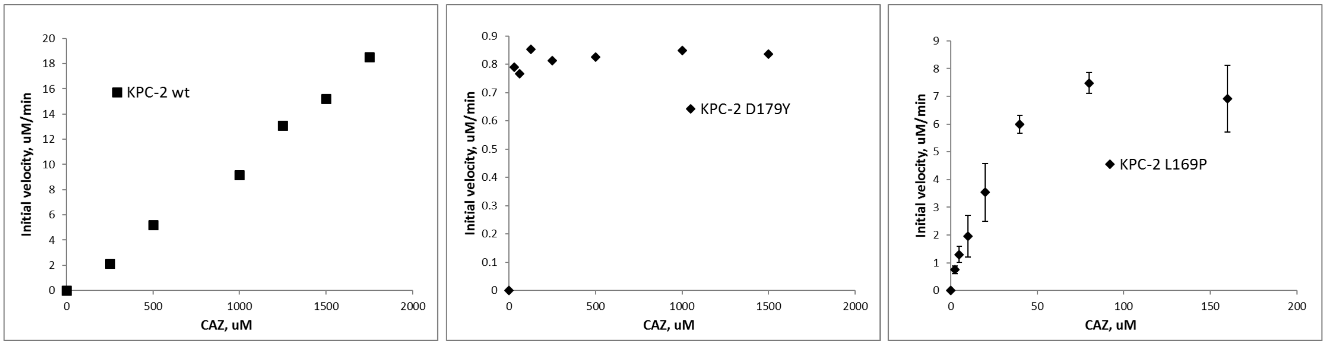

Protein preparations were mixed with various concentrations of ceftazidime and absorbance at 260 nm was monitored using spectrophotometer. Initial rates of substrate cleavage were calculated and plotted vs ceftazidime concentration

Figure S3. Kinetic profiles of KPC-2 wild type and L169P inactivation by avibactam and vaborbactam.

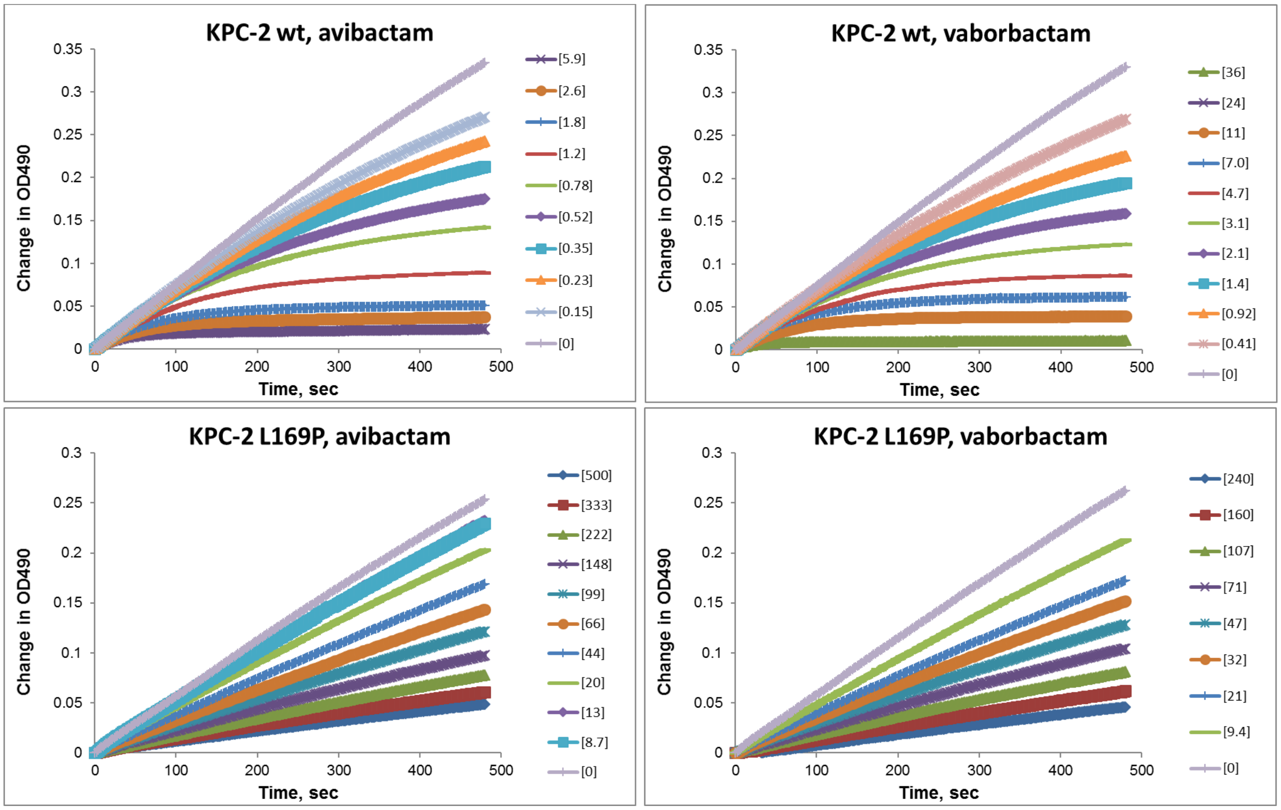

BLIs at indicated concentration (in µM) were quickly mixed with enzyme and 100 µM NCF as reporter substrate and absorbance at 490 nm was recorded immediately every 2 sec using plate reader

Figure S4. Kinetic profiles of KPC-2 D179Y inactivation by avibactam and vaborbactam.

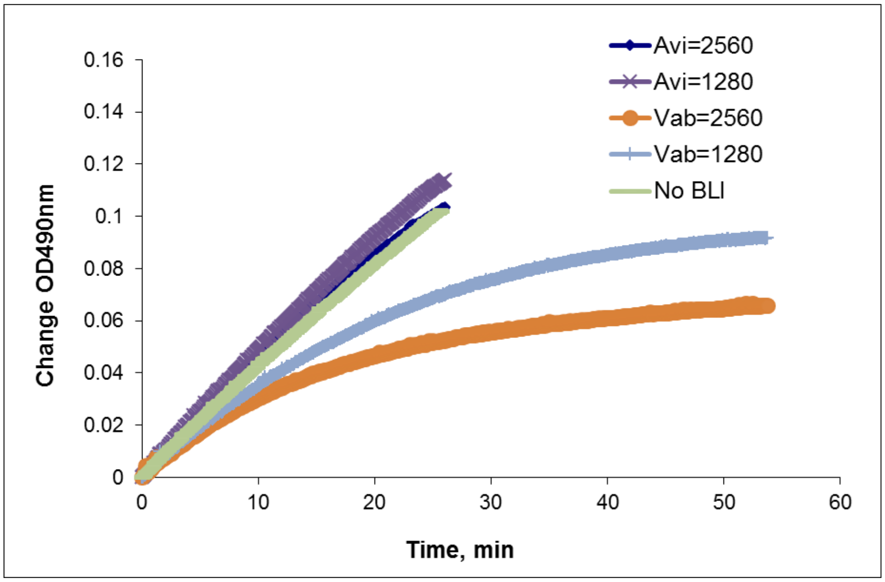

Kinetic profiles of KPC-2 D179Y inactivation by avibactam and vaborbactam. BLIs at indicated concentration (in μM) were quickly mixed with enzyme and 100 μM NCF as reporter substrate and absorbance at 490 nm was recorded immediately every 10 sec using plate read
